# A multicenter evaluation of a novel microfluidic rapid AST assay for Gram-negative bloodstream infections

**DOI:** 10.1128/jcm.00458-24

**Published:** 2024-09-26

**Authors:** Benjamin Berinson, Emma Davies, Jessie Torpner, Linnea Flinkfeldt, Jenny Fernberg, Amanda Åman, Johan Bergqvist, Håkan Öhrn, Jonas Ångström, Cecilia Johansson, Klara Jäder, Helena Andersson, Ehsan Ghaderi, Maria Rolf, Martin Sundqvist, Holger Rohde, Teresa Fernandez-Zafra, Christer Malmberg

**Affiliations:** 1 Institute of Medical Microbiology, Virology and Hygiene, University Medical Center Hamburg-Eppendorf, Hamburg, Germany; 2 Gradientech AB, Uppsala, Sweden; 3 Department of Clinical Microbiology, Uppsala University Hospital, Uppsala, Sweden; 4 Department of Laboratory Medicine, Clinical Microbiology, Örebro University Hospital, Örebro, Sweden; 5 Department of Laboratory Medicine, Clinical Microbiology, Faculty of Medicine and Health, Örebro University, Örebro, Sweden; 6 Department of Medical Sciences, Uppsala University, Uppsala, Sweden; Johns Hopkins University, Baltimore, Maryland, USA

**Keywords:** rapid AST, sepsis, diagnostics, multicenter study

## Abstract

Common phenotypic methods for antimicrobial susceptibility testing (AST) of bacteria are slow, labor intensive, and display considerable technical variability. The QuickMIC system provides rapid AST using a microfluidic linear gradient. Here, we evaluate the performance of QuickMIC at four different laboratories with regard to speed, precision, accuracy, and reproducibility in comparison to broth microdilution (BMD). Spiked (*n* = 411) and clinical blood cultures (*n* = 148) were tested with the QuickMIC Gram-negative panel and compared with BMD for the 12 on-panel antibiotics, and 10 defined strains were run at each site to measure reproducibility. Logistic and linear regression analysis was applied to explore factors affecting assay performance. The overall essential agreement and categorical agreement between QuickMIC and BMD were 95.6% and 96.0%, respectively. Very major error, major error, and minor error rates were 1.0%, 0.6%, and 2.4%, respectively. Inter-laboratory reproducibility between the sites was high at 98.9% using the acceptable standard of ±1 twofold dilution. The mean in-instrument analysis time overall was 3 h 13 min (SD: 29 min). Regression analysis indicated that QuickMIC is robust with regard to initial inoculum and delay time after blood culture positivity. We conclude that QuickMIC can be used to rapidly measure MIC directly from blood cultures in clinical settings with high reproducibility, precision, and accuracy. The microfluidics-generated linear gradient ensures high reproducibility between laboratories, thus allowing a high level of trust in MIC values from single testing, at the cost of reduced measurement range compared to BMD.

**IMPORTANCE:**

Increasing antimicrobial resistance underscores the need for new diagnostic solutions to guide therapy, but traditional antimicrobial susceptibility testing (AST) is often inadequate in time-critical diseases such as sepsis. This work presents a novel and rapid AST system with a rapid turnaround of results, which may help reduce the time to guided therapy, possibly allowing early de-escalation of broad-spectrum empirical therapy as well as rapid adjustments to treatments when coverage is lacking.

## INTRODUCTION

Antimicrobial resistance is a global public health concern, posing significant challenges to effective infection management. The inappropriate use of antibiotics contributes to the emergence and spread of resistant pathogens, leading to increased morbidity, mortality, and healthcare costs (1). Diagnostic interventions such as bacterial identification, syndromic testing, and especially antimicrobial susceptibility testing (AST) play a critical role in guiding appropriate antibiotic therapy, especially in critical diseases such as bloodstream infection (2). Mortality rates due to bloodstream infection range between 12% and 32% in North America and Europe and are even higher in low-income and middle-income countries (3, 4). Mortality is due in part to increasing rates of antimicrobial-resistant pathogens, and patients infected with resistant pathogens are more likely to receive ineffective empiric antibiotic therapy, which is associated with poor outcomes, including death (5, 6). Conversely, treatment with overly broad antibiotics increases the risk of adverse drug events and drives further development of resistance (7). Despite advances in antimicrobial susceptibility testing, the delays associated with standard of care tests can lead to suboptimal patient outcomes, increased length of hospital stays, and prolonged exposure to broad-spectrum antibiotics. Rapid antimicrobial susceptibility testing (rapid AST or rAST) offers the potential to overcome these challenges by providing timely and accurate results, allowing for prompt adjustment of antibiotic therapy and targeted treatment selection (4). Newly developed tools, like the Pheno system (Accelerate Diagnostics) or Vitek Reveal (bioMérieux), offer rAST results in 7 h and 5.5 h, respectively. EUCAST RAST disk diffusion can deliver even faster AST results (as early as 4 h), at a low cost, and has proven to be a valuable tool (8–10), but does not provide quantitative MIC values, is fairly laborious with exact intervals and manual reading [although automation is possible (11, 12)], as well as only providing breakpoints for a selection of species and antibiotic agents.

QuickMIC is an *in vitro* diagnostic system for rapid AST directly from positive blood cultures (PBCs). The technology is based on microfluidics and solid phase cytometry, providing growth-based susceptibility results in 2–4 h (13, 14). A linear antibiotic concentration gradient is generated in a 3D agarose hydrogel containing bacteria from the patient’s blood culture sample. The MIC can be rapidly identified via automated, real-time quantification of bacterial microcolony growth. The system consists of single-use, disposable QuickMIC cassettes with pre-filled, dried antibiotics, a modular QuickMIC Instrument to process the cassettes, and QuickMIC Analyst software for automated analysis. Instruments can be stacked for increased capacity (12 can be run by a single PC). One Instrument can analyze one patient sample against a panel of 12 antibiotics per run.

The aim of the current study was to evaluate the accuracy and precision of the QuickMIC system, the reproducibility between laboratories, identify performance limitations as well as to gain insight into the performance of the system in real-life microbiological workflows.

## MATERIALS AND METHODS

### Study locations

The data were collected from February to November 2022 at four separate locations; three clinical microbiological laboratories and the internal laboratory at Gradientech. The internal laboratory ran spiked samples and performed the broth microdilution (BMD) reference testing together with Eurofins Pegasuslab AB, Uppsala, Sweden (about 25% of tests). The three clinical laboratories were located in Uppsala and Örebro, Sweden, as well as Hamburg, Germany (Table 1). Of note, the clinical laboratories were not operated 24 h per day. At the clinical sites, blood cultures with Gram-negative pathogens were run on the QuickMIC system with the inclusion criteria being Gram-negative monomicrobial presentation under the microscope. From each individual patient, only one sample was included in the study. Confirmed polymicrobial samples and species not covered by the QuickMIC manufacturer’s instructions were excluded from the analysis.

**TABLE 1:** Details of the study locations^*a*^

	Gradientech	Örebro University Hospital	Uppsala University Hospital	University Medical Center Hamburg-Eppendorf
Location	Uppsala, Sweden	Örebro, Sweden	Uppsala, Sweden	Hamburg, Germany
Size (no. of beds)	–^*b*^	Small (460)	Medium (850)	Large (1,700)
Blood culture sets/year	–	20,000 BCs	27,000 BCs	25,000 BCs
Blood culture system	Bactec FX40 (BD)	Bactec FX (BD)	Bact/Alert Virtuo (bioMérieux)	Bactec FX (BD)
Bacterial ID method	MALDI-ToF	MALDI-ToF (from positive BC vial)	MALDI-ToF [from shortly incubated agar plate (4 h)]	MALDI-ToF [from shortly incubated agar plate (4 h)]
Samples tested	Challenge, reproducibility	Clinical, reproducibility	Clinical, reproducibility	Clinical, reproducibility
S samples (%)	37.2	80.4	75.0	70.3
R samples (%)	62.8	19.6	25.0	29.7
MDR samples (%)	40.9	3.9	5.0	5.4
No. of samples in study (%)	411 (73.5)	51 (9.1)	37 (6.6)	60 (10.8)

^*a*^ S, susceptible to all antibiotics tested; R, resistant to at least one tested antibiotic; MDR, resistant to at least three different classes of antibiotics; MALDI-ToF, matrix-assisted laser desorption/ionization time-of-flight mass spectrometer.

^*b*^ –, not applicable.

### QuickMIC testing with spiked challenge isolates

Accuracy evaluation was performed with a reference collection of 411 bacterial challenge isolates with high resistance rates, acquired from multiple sources (Table S1). The isolates were chosen to include a wide variety of susceptibility profiles for each antibiotic on the QuickMIC Gram-negative (GN) panel. The species distributions are presented in Table 2.

**TABLE 2:** Species distribution of tested isolates

	*Citrobacter* spp.	*Enterobacter cloacae* complex	*Proteus* spp.	*Pseudomonas aeruginosa*	*Serratia marcescens*	*Klebsiella* spp.	*Escherichia coli*	*Acinetobacter baumannii* complex	Total
Clinical	3	3	4	6	8	28	96	0	148
Challenge	15	29	19	58	13	129	128	20	411
Total	18	32	23	64	21	157	224	20	559
Reproducibility	0	2	0	2	0	3	3	0	10

The bacterial isolates were streaked on agar plates, grown overnight, and the next day harvested into freezing buffer. The isolates were kept frozen at −70°C for the remainder of the study. All isolates were cultivated using Müller-Hinton (MH) II-agar or MH-II broth (BBL, Becton Dickinson).

Spiked blood cultures for QuickMIC testing were prepared by streaking frozen isolates on agar plates and incubating overnight at 37°C. The next day, 2–4 colonies were resuspended in MH-II broth, adjusted to 0.5 McFarland, and diluted to a target concentration of 100 CFU/mL into human donor blood or citrated horse blood, after which 10 mL of the blood mixture was used to inoculate blood culture bottles (Bactec Plus-Aerobic/F Medium, BD), resulting in a start concentration in the blood bottles of 25 CFU/mL. The spiked blood culture bottles were then incubated in a Bactec FX40 system (BD) until a positive signal was received. After positivity, bottles were removed for QuickMIC testing using the QuickMIC GN cassettes (art. nr: 43001). QuickMIC testing was performed as per the protocol of the manufacturer. In brief, ca. 10 µL of PBC is added to a sample vial using a standard inoculation loop. The vial contents are filtered to remove cells and debris and then injected into the cassette. The gel is allowed to solidify for 15 min and afterward, the cassette is loaded into the instrument.

### QuickMIC testing of reproducibility isolates

For measuring the precision and reproducibility of the QuickMIC system, 10 isolates of the reference collection were sent to every location and named RP1–RP10 (Table 2). The isolates were prepared for QuickMIC using the manufacturer’s protocol for running isolated colonies from plate. In brief, the bacteria were streaked onto an agar plate, incubated overnight at 37°C, and resuspended to 0.5 McFarland in PBS, and 10 µL of this solution was used instead of positive blood culture, otherwise using the standard QuickMIC workflow. The 10 isolates were run in at least triplicates (on different days) at each study site. Reproducibility was calculated as the total number of results that span a maximum of three twofold dilutions divided by the total number of results, overall study sites. MIC data expressed in a bi-logarithmic scale were used, and only antibiotic-bacteria combinations with applicable breakpoints according to EUCAST guidelines (v 13.0, 2023, available at www.eucast.org) were analyzed. Precision of linear MIC results was calculated as the median standard deviation (SD) of all results for each antibiotic-drug combination, with the results for each strain normalized to the maximum antibiotic concentration.

### QuickMIC testing of clinical samples

For patient samples, positive blood cultures were handled as per the routine protocol at each hospital laboratory, and samples were run on the QuickMIC system using QuickMIC GN cassettes according to the standard protocol of the manufacturer. The samples were further subcultured, identified, and handled according to the routine workflow at each hospital laboratory. Bacterial isolates were prepared from the subculture plates and shipped to Gradientech for BMD reference testing. Collected isolates were stored at −70°C throughout the study.

### Broth microdilution reference testing

Broth microdilution was performed according to ISO 20776-1:2019 [International Organization for Standardization (ISO)] using pre-filled plates (Merlin Diagnostika GmbH, Bornheim-Hersel, Germany). The antibiotics, antibiotic abbreviations, and concentrations used are described in Table S2. In brief, the plates were inoculated using a bacterial suspension at 0.5 McFarland prepared from pure colonies from a solid agar medium, yielding a final concentration of ~5 × 10^5^ CFU/mL per well, and incubated overnight at 37°C. After 18–20 h, the MIC was determined according to guidelines in the EUCAST reading guide for broth microdilution v 4.0 (available at www.eucast.org). All isolates were tested at least twice on different days. If the results did not agree, a further replicate was tested, and the modal result was used. If no modal MIC could be determined, a further replicate was tested. If no modal MIC could be achieved after five replicate tests, the MIC value was reported as “not available.” The reproducibility isolates (RP) were tested up to 14 times using different inoculates and by different operators to quantify the variability in the reference BMD method.

### Data analysis

The QuickMIC AST results, date and time of blood culture start, positivity, and unloading, as well as the start and end times of QuickMIC testing were recorded. QuickMIC MIC results generated by the Analyst software (v 1.0.7) were compared with reference results as previously described (13). Briefly, linear-scale MIC values were automatically right-censored to the nearest twofold dilution in an equivalent BMD assay, and essential agreement (EA) (results within ±1 twofold dilution from reference) and bias were calculated as per ISO 20776-2:2021. Discrepancy resolution or retesting in case of non-agreement was not used in this study. When the reference method showed results below or above the limit of quantification (LOQ) for QuickMIC, these were truncated to the QuickMIC range, as specified by ISO 20776-2:2021. Categorical agreement (CA) (results differing in interpreted category) was calculated after categorization by applying EUCAST clinical breakpoints (v 13.0, 2023, available at www.eucast.org). The test results outside of categorical agreement were categorized by error type, and the fraction of very major error (VME), major error (ME), and minor error (mE) results were calculated using the denominator of the entire data set, so that fraction of categorical agreement, VME, ME, and mE always add up to 100. Furthermore, the VME and ME error rates were calculated by using the number of resistant isolates (VME) and susceptible isolates (ME) as denominator. Linear or logistic regression was performed in R (v 4.3.1) to investigate parameters influencing the result quality, depending on the type of outcome variable. For analyzing effects on agreement, logistic regression was used, whereas continuous outcome variables, such as analysis time, were analyzed using linear regression. For reproducibility analysis, the mean MIC and SD of MIC of all runs were calculated, and the SD normalized to the maximum concentration for each tested QC strain and antibiotic.

## RESULTS

### Reproducibility of QuickMIC between laboratory sites

**Fig 1:**
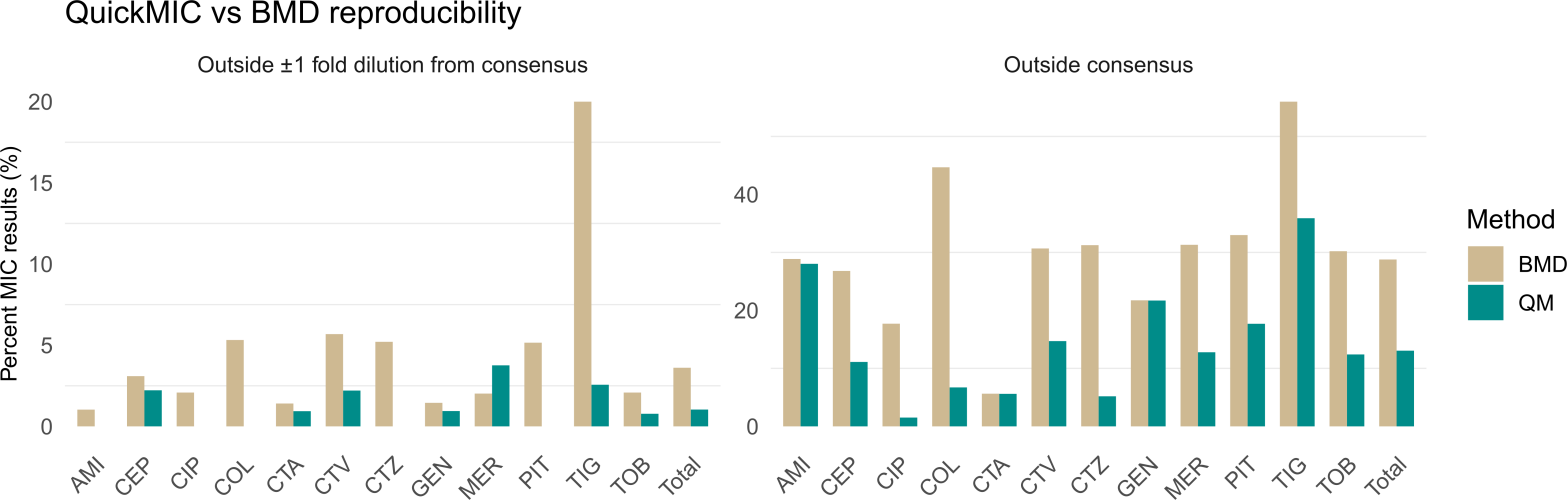
Reproducibility of QuickMIC (green) compared to BMD (brown) showing percent of results within one twofold dilution (left) for each antibiotic, and percent of all results not yielding exactly the consensus MIC for each antibiotic. QuickMIC is overall more reproducible.

The overall reproducibility was 98.9%. Results were within one twofold dilution between the sites, calculated from the twofold dilution QuickMIC result. In total, 36% of all data points were within the measurement range. The precision of the linear MIC value was also evaluated and calculated as the standard deviation between linear MIC replicates. For the complete data set, the overall precision (median standard deviation) was 3.0%, and ranged between 1.3% and 8.6% per included antibiotic (Table 3). The variation in the twofold MIC value was further compared to the variation in the BMD method (Fig. 1; Fig. S1), indicating that the QuickMIC sample-sample variation was lower than BMD. On average for all antibiotics, 29% vs 13% of all replicates deviated from the consensus MIC using the BMD method and QuickMIC, respectively, corresponding to an overall 2.2× higher reproducibility (range 1–11× higher, per antibiotic).

**TABLE 3:** Results from reproducibility and precision testing per antibiotic^*a*^

Antibiotic^*b*^	Number of data points within measurement range	Number of data points within ±1 twofold dilution	Total number of data points	Reproducibility (%)	Precision (median SD of linear MIC in %)
AMI	94	127	127	100	4.3
CEP	35	128	131	97.7	3.2
CIP	38	132	132	100	3.0
COL	17	120	120	100	1.3
CTA	25	106	107	99.1	5.3
CTV	38	125	128	97.7	3.0
CTZ	40	133	133	100	3.4
GEN	44	105	106	99.1	2.1
MER	33	110	115	95.7	8.6
PIT	50	124	124	100	1.9
TIG	21	38	39	97.4	3.0
TOB	60	99	100	99	5.4
Total	495	1,347	1,362	98.9	3.0

^*a*^ The table summarizes the reproducibility (number of data points within allowable variation as fraction of all data points), as well as the precision of the continuous scale linear MIC, as median SD of the MIC for each antibiotic-bacteria target normalized to each maximum concentration tested.

^*b*^ Antibiotic abbreviations follow the EUCAST system for antibiotic abbreviations, v 7 (Table S2).

### QuickMIC performance on challenge and clinical isolates

The challenge isolate collection consisted of a selection of common as well as more rarely encountered species in positive blood cultures with a comparatively large amount of resistant isolates (62.8% of all isolates having at least one resistance on the tested panel, compared to 24.3% for the clinical data set). All the species included in the study and their respective numbers are depicted in Table 2. The overall essential agreement, categorical agreement, and errors for the challenge isolates are presented in Table 4. Performance per antibiotic was overall acceptable at >90% EA, CA, and bias within ±30%, except for meropenem and piperacillin/tazobactam. The QuickMIC system produced MIC results within 2–4 h, excluding sample preparation. For the clinical samples, 148 samples were collected from the three locations, representing most species commonly encountered in bloodstream infections. A more detailed breakdown of the susceptibility profiles for each species and each antibiotic is shown in Fig. S2. The time from the start of the incubation of the blood culture bottles to QM result (total turnaround time) was on average 27.4 h for the clinical samples, and the time from blood culture unload to AST result (turnaround time, TAT) was on average 9.2 h (SD: 4 h) (Table S3 ; Fig. S3A). CA and EA were overall >90% for all tested antibiotics compared to BMD (Table 5). No systematic differences could be seen between challenge and clinical isolates. QuickMIC performance for the complete data set (clinical and challenge data sets together, Table 6) had an EA and CA >90% for all tested antibiotics, and average time to result of 3 h and 17 min. When examined by Enterobacterales vs non-fermenters (*P. aeruginosa* and *A. baumannii*), both EA and CA were higher for Enterobacterales, although EA and CA were >90% compared to BMD for both species groupings (Tables 7 and 8). For non-fermenters (Table 8), several antibiotics were <90% EA and CA, but most discrepancies were minor except for ceftazidime/avibactam with a high fraction of major errors.

**TABLE 4:** Results for spiked challenge isolates

	N_results_	CA	EA	S	I	R	mE	ME (%/%_S_)^*a*^	VME (%/%_R_)^*a*^	Bias	Mean time (h:min)
AMI	345	333 (96.5)	315 (91.3)	297	0	48	0 (0)	2 (0.6/0.7)	10 (2.9/20.8)	14.73	2:34
CEP	312	281 (90.1)	287 (92)	173	20	119	26 (8.3)	1 (0.3/0.6)	4 (1.3/3.4)	−20.50	3:27
CIP	378	365 (96.6)	372 (98.4)	170	36	172	13 (3.4)	0 (0)	0 (0)	−7.26	3:32
COL	289	283 (97.9)	279 (96.5)	232	0	57	0 (0)	0 (0)	6 (2.1/10.5)	−25.03	3:04
CTA	309	305 (98.7)	307 (99.4)	159	7	143	4 (1.3)	0 (0)	0 (0)	−4.70	3:36
CTV	330	324 (98.2)	320 (97)	292	0	38	0 (0)	6 (1.8/2.1)	0 (0)	−15.70	3:12
CTZ	345	329 (95.4)	335 (97.1)	158	45	142	15 (4.3)	0 (0)	1 (0.3/0.7)	4.89	3:33
GEN	313	306 (97.8)	305 (97.4)	237	0	76	0 (0)	4 (1.3/5.3)	3 (1/3.9)	19.14	3:11
MER	362	315 (87)	322 (89)	280	25	57	35 (9.7)	0 (0)	12 (3.3/21.1)	−40.66	3:25
PIT	317	285 (89.9)	286 (90.2)	171	33	113	18 (5.7)	4 (1.3/2.3)	10 (3.2/8.8)	−17.83	3:30
TIG	114	114 (100)	112 (98.2)	113	0	1	0 (0)	0 (0)	0 (0)	–^*b*^	3:06
TOB	337	332 (98.5)	329 (97.6)	227	0	110	0 (0)	2 (0.6/0.9)	3 (0.9/2.7)	29.84	3:07
Overall	3,751	3,572 (95.2)	3,569 (95.1)	2,509	166	1,076	111 (3)	19 (0.5/0.8)	49 (1.3/4.6)	−4.52	3:17

^*a*^ %: error fraction of whole dataset, %_S_: error fraction of susceptible isolates only (ME rate), %_R_: error fraction of resistant isolates only (VME rate).

^*b*^ –, not evaluable; too few results within measurement range.

**TABLE 5:** Results for clinical isolates

	N_results_	CA	EA	S	I	R	mE	ME (%/%_S_)	VME (%/%_R_)	Bias	Mean time (h:min)
AMI	138	137 (99.3)	128 (92.8)	137	0	1	0 (0)	1 (0.7/0.7)	0 (0)	22.02	2:26
CEP	142	134 (94.4)	134 (94.4)	132	5	5	7 (4.9)	1 (0.7/0.8)	0 (0)	−30.25	3:12
CIP	138	129 (93.5)	135 (97.8)	114	10	14	8 (5.8)	1 (0.7/0.9)	0 (0)	−9.72	3:14
COL	127	127 (100)	125 (98.4)	115	0	12	0 (0)	0 (0)	0 (0)	−34.33	2:59
CTA	131	130 (99.2)	130 (99.2)	122	1	8	0 (0)	1 (0.8/0.8)	0 (0)	−14.96	3:23
CTV	142	141 (99.3)	140 (98.6)	142	0	0	0 (0)	1 (0.7/0.7)	0 (0)	−15.77	2:55
CTZ	143	138 (96.5)	138 (96.5)	128	8	7	3 (2.1)	2 (1.4/1.6)	0 (0)	−5.88	3:16
GEN	117	114 (97.4)	111 (94.9)	112	0	5	0 (0)	3 (2.6/2.7)	0 (0)	30.89	3:01
MER	138	138 (100)	137 (99.3)	138	0	0	0 (0)	0 (0)	0 (0)	2.17	3:06
PIT	134	129 (96.3)	128 (95.5)	123	5	6	1 (0.7)	1 (0.7/0.8)	3 (2.2/50.0)	−29.95	3:15
TIG	82	82 (100)	79 (96.3)	82	0	0	0 (0)	0 (0)	0 (0)	*–^a^*	3:03
TOB	125	123 (98.4)	118 (94.4)	120	0	5	0 (0)	2 (1.6/1.7)	0 (0)	29.52	2:53
Overall	1,557	1,522 (97.8)	1,503 (96.5)	1,465	29	63	19 (1.2)	13 (0.8/0.9)	3 (0.2/4.8)	−14.41	3:04

^*a*^ –, not evaluable; too few results within measurement range.

**TABLE 6:** Summary of overall results (clinical and spiked challenge data sets)

	AMI	CEP	CIP	COL	CTA	CTV	CTZ	GEN	MER	PIT	TIG	TOB	Overall
CA (%)	97.3	91.4	95.7	98.6	98.9	98.5	95.7	97.7	90.6	91.8	100	98.5	96.0
EA (%)	91.7	92.7	98.3	97.1	99.3	97.5	96.9	96.7	91.8	91.8	97.4	96.8	95.6
mE (%)	0.0	7.3	4.1	0.0	0.9	0.0	3.7	0.0	7.0	4.2	0.0	0.0	2.4
ME (%/%_S_)	0.6/0.7	0.4/0.7	0.2/0.4	0.0/0.0	0.2/0.4	1.5/1.6	0.4/0.7	1.6/2.0	0.0/0.0	1.1/1.7	0.0/0.0	0.9/1.2	0.6/0.8
VME (%/%_R_)	2.1/20.4	0.9/3.2	0.0/0.0	1.4/8.7	0.0/0.0	0.0/0.0	0.2/0.7	0.7/3.7	2.4/21.1	2.9/10.9	0.0/0.0	0.6/2.6	1.0/4.6
Result time (h:min)	2:32	3:23	3:27	3:03	3:32	3:07	3:28	3:08	3:20	3:26	3:05	3:03	3:13

**TABLE 7:** Summary of overall results (Enterobacterales)

	AMI	CEP	CIP	COL	CTA	CTV	CTZ	GEN	MER	PIT	TIG	TOB	Overall
CA (%)	98.1	93.4	95.7	98.6	98.9	99.3	96.5	97.6	91.8	92	100	98.7	96.5
EA (%)	91.5	93.6	98.4	97.6	99.3	98	97.9	96.8	92	92	97.4	97.7	96
mE (%)	0	5.2	4.1	0	0.9	0	2.8	0	5.7	3.6	0	0	2
ME (%/%_S_)	0.7/0.8	0.5/0.7	0.2/0.4	0/0	0.2/0.4	0.7/0.7	0.5/0.7	1.7/2.1	0/0	1.2/1.7	0/0	0.8/1.0	0.6/0.7
VME (%/%_R_)	1.2/14.7	0.9/3.7	0/0	1.4/7.8	0/0	0/0	0.2/0.7	0.7/4.2	2.5/29.7	3.2/12.3	0/0	0.5/2.1	0.9/4.6
Result time (h:min)	02:30	03:21	03:24	03:00	03:32	03:05	03:25	03:08	03:16	03:24	03:05	03:03	03:12

**TABLE 8:** Summary of overall results (non-fermenters)

	AMI	CEP	CIP	COL	CTV	CTZ	GEN	MER	PIT	TOB	Overall
CA (%)	93	64.5	96.2	97.8	85.7	89.1	100	81.7	89.7	97.2	90.6
EA (%)	93	80.6	97.4	93.5	89.3	89.1	95	90	89.7	91.5	91.6
mE (%)	0	35.5	3.8	0	0	10.9	0	16.7	10.3	0	6.8
ME (%/%_S_)	0/0	0/0	0/0	0/0	14.3/22.2	0/0	0/0	0/0	0/0	1.4/2	1/2.4
VME (%/%_R_)	7	0	0	2.2	0	0	0	1.7/5.0	0/0	1.4/4.8	1.6/4.5
Result time (h:min)	02:46	03:48	03:45	03:26	03:46	03:50	03:22	03:45	03:50	03:02	03:29

### Regression analysis of parameters influencing performance

**Fig 2:**
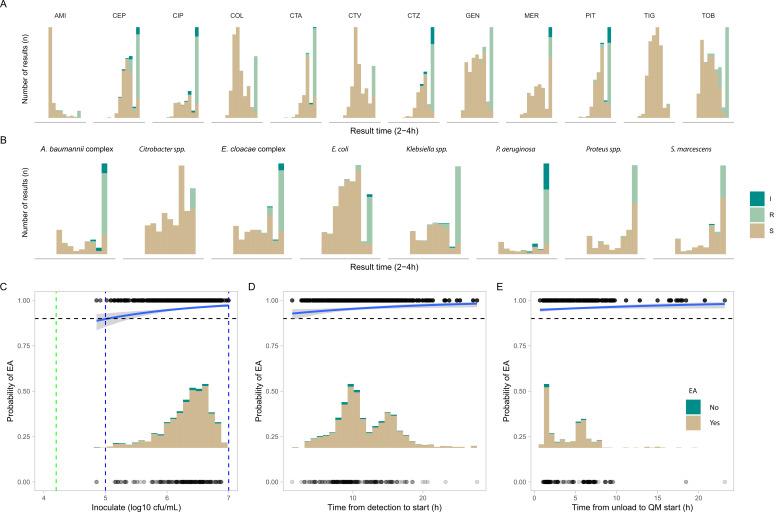
**(A and B**) Distribution of time to MIC for all results, split over antibiotics and species analyzed, indicating trends in the analysis time dependence on susceptibility category, where R/I results take longer time to result than S results. (**C–E**) Logistic and linear regression analysis of parameters affecting QuickMIC performance. (**C**) The ranges of inoculates achieved after sample preparation in comparison to likelihood of achieving accurate results (within EA) from the logistic regression analysis. Blue dashed lines indicate limits of quantitation, where estimated likelihood of EA > 90%, gray field is 95% confidence interval. The green dashed line indicates limit of detection, the lowest limit of quantifiable inoculum density. (**D and E**) display the same analysis for the time from blood culture detection to AST start and time from blood culture bottle unload to AST start, none of which are significantly affecting performance. The black dashed line indicates the limit of acceptable performance at 90% probability of achieving essential agreement, where the scale 0–1 represents 0%–100% probability.

Logistic and linear regression analyses were used to investigate factors influencing QuickMIC result accuracy and analysis time (Fig. 2). Parameters investigated were reference susceptibility category, species, antibiotics, inoculate concentration of the starting blood culture, and delay times from culture bottle positivity and culture bottle unload until the start of AST analysis. Resistance category, species, and antibiotic were found to significantly affect analysis time, with resistant isolates taking on average 38 min longer (*P* < 0.001) to final result than susceptible ones (Fig. S3B). An overview of the distribution of analysis times over species and antibiotics is displayed in Fig. 2A and B. Regarding effects on accuracy, time from positivity until start of analysis as well as time from unload to start of analysis were not significant factors in the time range encompassing 0–20 h (*P* = 0.16, *P* = 0.37, respectively) (Fig. 2D and E). Starting inoculum after the sample preparation step had a significant effect on result accuracy, however (*P* < 0.001), with start inoculum concentration <1 × 10^5^ estimated to provide unacceptable performance (overall estimated EA < 90%, Fig. 2C). Considering this cutoff, 556 out of 559 (99.5%) of all tested blood cultures provided an inoculum within an acceptable performance range after sample preparation.

## DISCUSSION

The data presented here show that the QuickMIC rapid AST system has overall good accuracy compared to the reference method broth microdilution with a significantly improved reproducibility. The three clinical laboratories taking part in the study represented a range of small to large hospital settings and two of the most common blood culture systems in use today, showing wide applicability of the method. The results were of high accuracy also for the challenge data set, containing a high rate of resistant and multidrug-resistant isolates which can be difficult for rapid AST methods to handle due to, e.g., delayed resistance (15). Of note, EA and/or CA for meropenem and piperacillin/tazobactam were lower than 90%, specifically in the challenge data set, reflecting difficulties with these antibiotics. Piperacillin/tazobactam is known to be a problematic antibiotic for many AST methods (16, 17). There are several reasons hypothesized, both relating to bacterial genetics and heteroresistance, as well as stability issues with the antibiotic itself. Another issue is related to the breakpoints, where piperacillin/tazobactam breakpoints lack the I category, and the S/R breakpoint is positioned directly next to the epidemiological cut-off (“wild-type” population). This means that very small errors, even MIC results that are inside essential agreement with the reference method, may result in major or very major categorical errors for strains with MIC close to the breakpoint. However, the bias was high for piperacillin/tazobactam (−17.83%) and outside the recommended 30% for meropenem, meaning a systematic undercalling of the meropenem MIC, which will be investigated further. Adjustments to cassette manufacturing and the analysis algorithm may be necessary to improve the bias. Strikingly, 8/12 VME for meropenem were isolates from the CDC AR isolate bank. For the CDC strains, the genetic information on acquired resistance genes is available; however, a brief analysis did not yield any obvious pattern among the meropenem VME results, with three KPC-type, one VIM-type, two NDM-type, and two OXA-type carbapenemases among the eight CDC isolates. Further analysis on whether specific acquired resistance genes are over-represented in isolates with poor carbapenem rapid AST performance is currently ongoing and falls outside the scope of this study.

Several rAST systems have emerged on the market in recent years in part due to a concerted effort from the public sector involving building awareness as well as economic push and pull incentives (18–20). Examples include Pheno (Accelerate Diagnostics), dRAST (Quantamatrix), ASTar (Q-Linea), Reveal (bioMérieux, formerly Specific Bioscience) as well as the RAST method from EUCAST. These methods overall display good accuracy >90% and average time to result of 4–7 h, which is markedly slower than the 2–4 h presented here. The precise, microfluidically generated linear gradient used in QuickMIC is the main differentiator to other rapid AST technologies available today. The exact gradient (13) allows for high reproducibility and precise MIC results, as demonstrated here. The clinical value of the added precision from a linear MIC, with a linear measurement error, resulting in a more precise MIC with less error range, remains to be explored. However, the increased precision also manifests in the reduced variability in the twofold MIC result demonstrated here, which may be valuable for example in situations where the MIC is close to the clinical breakpoint. Higher reproducibility may offer an advantage since laboratories often perform single-sample testing due to cost. Greater confidence in MIC values might also lead to novel applications in areas such as precision dosing and personalized medicine in infectious diseases, including PK/PD optimized dosing approaches incorporating the MIC in dosage adjustment (21–23). MIC-based dosage adjustment as a concept has been criticized due to the high inherent variability of existing AST methods (24), a problem that potentially could be rectified by using a more precise AST system. The possibilities opened by a rapid and more precise MIC method warrant further studies on these matters.

**Fig 3:**
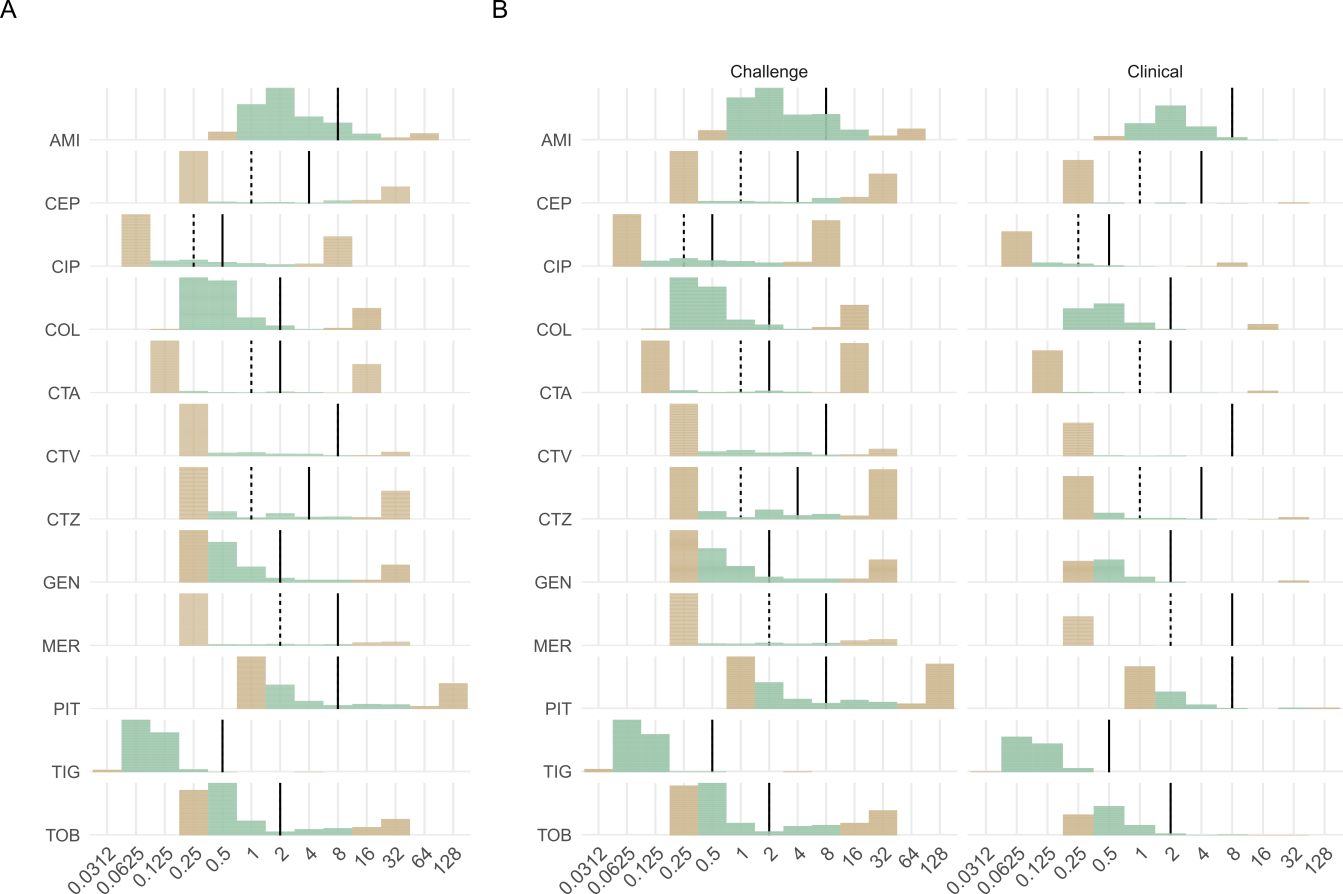
Distribution of MIC results from all strains included in the study. Color coding according to reference MIC being within QuickMIC’s measurement range or not: green = in QuickMIC measurement range. Lines indicate I/R breakpoints (I = dashed, R = solid). (A) depicts the combined results from the clinical and spiked samples while (B) shows split by clinical or spiked sample collection.

The tradeoff of using a linear gradient is reduced range in comparison to dilution-based methods. In this data set, 31.5% of all QuickMIC results were within the measurement range (59.0% for BMD). The LOQ of QuickMIC is 5% of the maximum measurable concentration, which translates to a range covering approximately five twofold dilutions, compared to the larger range of 8–12 twofold dilutions that is covered by the BMD method. In practice, the QuickMIC measurement range is set to include the clinical breakpoints, with either one or two twofold dilutions above the R breakpoint or below the S breakpoint (Fig. 3). EUCAST emphasizes that measuring MIC in the low breakpoint regions is of limited clinical use (25), which reduces the implications for this limitation in range. Similar arguments can be made for the extremely high resistance range.

The inclusion of hospitals only from low to middle resistance settings, in Northern and Central Europe is a main limitation of this study. It is clear from the performance data that the susceptibility category of the tested isolates has an impact on accuracy and result time. Even though this study tries to counter this bias by inclusion of a highly resistant challenge data set, convincing data from clinical real-life samples in a high resistance setting are important and need to be investigated further. Other limitations include the handling of polymicrobial samples, which are not supported by the QuickMIC system and have been excluded from analysis here.

Finally, it should be noted that while the current ISO 20776-2:2021 standard has removed the performance limits on categorical agreement and error rates, the QuickMIC system does in some cases exceed the error rate limits as defined by the previous version of the standard. As an example, in this data set, piperacillin-tazobactam displays a VME rate of 11% using the deprecated definition (13 VME of 119 resistant tested strains); while still displaying EA and bias well within the acceptable range of >90% and −30 to +30%, respectively. Even though the bias value is now the mandated method for evaluating the performance of AST methods, these discrepancies should be kept in mind and investigated further. The reason for using bias instead of error rates is well-argued; however, since low bias and high EA by definition indicate high accuracy of the results as compared to the reference method, and bias is also immune to breakpoint changes and similar issues which complicate the use of CA and error rates for evaluating AST device performance. Furthermore, CA and error rates are sensitive to the composition of the data set, such as the number of resistant strains included, which is also the main argument behind removing the CA and error rate limits from the current standard.

This study demonstrates AST in 2–4 h directly from PBCs. However, it should be noted that the TAT for the clinical samples in the three locations averaged 9 h (SD: 4 h), which reflects the differences in PBC laboratory workflow. The impact of these procedural differences, manifesting in a delay time from blood culture unloading until AST start ranging from 41 min to 23 h, was investigated further by regression analysis. While we show that a wide range of delay times from culture positivity as well as from bottle unload to AST start are compatible with accurate rapid AST results, the delay times should be reduced as far as possible for maximum clinical impact of rapid AST.

In conclusion, QuickMIC can be used to rapidly measure MIC directly from blood cultures in clinical settings with high reproducibility, precision, and accuracy. The microfluidics-generated linear gradient ensures high reproducibility between laboratories, thus allowing a high level of trust in MIC values from single testing, at the cost of reduced measurement range compared to dilution-based methods. The impact on patient outcomes from this technology warrants further investigation, especially concerning the increased precision of the MIC values produced.

## Data Availability

The data sets presented in this study can be provided on request.

## SUPPLEMENTAL MATERIAL

Supplemental material jcm.00458-24-s0001.docx Tables S1 to S3; Figures S1 to S3.

ASM does not own the copyrights to Supplemental Material that may be linked to, or accessed through, an article. The authors have granted ASM a non-exclusive, world-wide license to publish the Supplemental Material files. Please contact the corresponding author directly for reuse.
