# Supplementary material for: A multicenter evaluation of a novel microfluidic rapid AST assay for Gram-negative bloodstream infections
Source: J Clin Microbiol. 2024 Sep 26;62(10):e00458-24. doi: 10.1128/jcm.00458-24 (PMC11481479; doi:10.1128/jcm.00458-24)
Supplement: Supplemental material — Tables S1 to S3; Figures S1 to S3. [file jcm.00458-24-s0001.docx]

# Supplementary data

A multicenter evaluation of a novel microfluidic rapid AST assay for Gram-negative bloodstream infections

Benjamin Berinson^1^, Emma Davies^2^, Jessie Torpner^2^, Linnea Flinkfeldt^2^, Jenny Fernberg^2^, Amanda Åman^2^, Johan Bergqvist^2^, Håkan Öhrn^2^, Jonas Ångström^2^, Cecilia Johansson^2^, , Klara Jäder^3^, Helena Andersson^3^, Ehsan Ghaderi^3^, Maria Rolf^4^, Martin Sundqvist^5^, Holger Rohde^1^, Teresa Fernandez-Zafra^2*^, Christer Malmberg^2,6*^

*^1^ Institute of Medical Microbiology, Virology and Hygiene, University Medical Center Hamburg-Eppendorf, Hamburg, Germany
^2^ Gradientech AB, Uppsala, Sweden*

*^3^ Department of Clinical Microbiology, Uppsala University Hospital, Uppsala, Sweden
^4^ Department of Laboratory Medicine, Clinical Microbiology, Örebro University Hospital, Örebro, Sweden*

*^5^ Department of Laboratory Medicine, Clinical Microbiology, Faculty of Medicine and Health, Örebro University, Örebro, Sweden
^6^ Department of Medical Sciences, Uppsala University, Uppsala, Sweden*

** These authors contributed equally to the study*

# Supplementary Tables

*Supplementary Table 1. Origin of challenge isolates.*

| **Origin** | **Number of isolates** |
| --- | --- |
| Alicante University General Hospital, Alicante, Spain | 18 |
| Antibiotic Research Unit, Uppsala University, Uppsala, Sweden | 125 |
| The AR ISOLATE BANK, CDC, USA (through the Public Health Agency of Sweden, Solna, Sweden) | 100 |
| The EUCAST Development Laboratory, Växjö, Sweden | 11 |
| University Clinic of Eppendorf, Hamburg, Germany | 23 |
| East Tallinn Central Hospital, Tallinn, Estonia | 17 |
| Uppsala University Hospital, Uppsala, Sweden | 94 |
| General University Hospital of Valencia, Valencia, Spain | 17 |

Supplementary Table 2. BMD reference panel and drug abbreviations.

| Antibiotic* | | Measuring range (mg/L) | EUCAST breakpoints (v13)  (Enterobacterales, S/R, mg/L) |
| --- | --- | --- | --- |
| AMI | Amikacin | 0.5-32 | 8/8 |
| CEP | Cefepime | 0.25-16 | 1/4 |
| CIP | Ciprofloxacin | 0.0625-4 | 0.25/0.5 |
| COL | Colistin | 0.125-8 | 2/2 |
| CTA | Cefotaxime | 0.125-8 | 1/2 |
| CTV | Ceftazidime/Avibactam | 0.25-16 | 8/8 |
| CTZ | Ceftazidime | 0.25-16 | 1/4 |
| GEN | Gentamicin | 0.25-16 | 2/2 |
| MER | Meropenem | 0.25-16 | 2/8 |
| PIT | Piperacillin/Tazobactam | 1-64 | 8/8 |
| TIG | Tigecycline | 0.0312-2 | 0.5/0.5 |
| TOB | Tobramycin | 0.25-16 | 2/2 |

## * Abbreviations according to the EUCAST system for antimicrobial abbreviation, v7

Supplementary Table 3. Summary of average time performance parameters from the different locations and sample types (clinical, challenge).

|  | Time until BC detection | BC load to AST end (TTAT) | BC detection to AST end | BC unload to AST end (TAT) | Analysis time in QuickMIC |
| --- | --- | --- | --- | --- | --- |
| Challenge dataset (Gradientech) | 9.3 (1.6) | 23.1 (2.7) | 13.6 (3.1) | 5.9 (2.7) | 3.3 (0.5) |
| Örebro University Hospital | 12.5 (20.3) | 26.4 (21.2) | 12.6 (6.4) | 8.4 (2.4) | 3.1 (0.4) |
| University Medical Center Hamburg-Eppendorf | 11.9 (24.5) | 29.9 (26.5) | 16.4 (6.6) | 10.4 (3.7) | 3.1 (0.4) |
| Uppsala University Hospital | 10.7 (13.5) | 27 (13.9) | 14.5 (5.3) | 9.1 (4.9) | 3 (0.4) |
| Overall clinical | 11.6 (19.2) | 27.4 (20.3) | 13.8 (6.2) | 9.2 (4) | 3.1 (0.4) |

Numbers in brackets indicate SD.

# Supplementary Figures


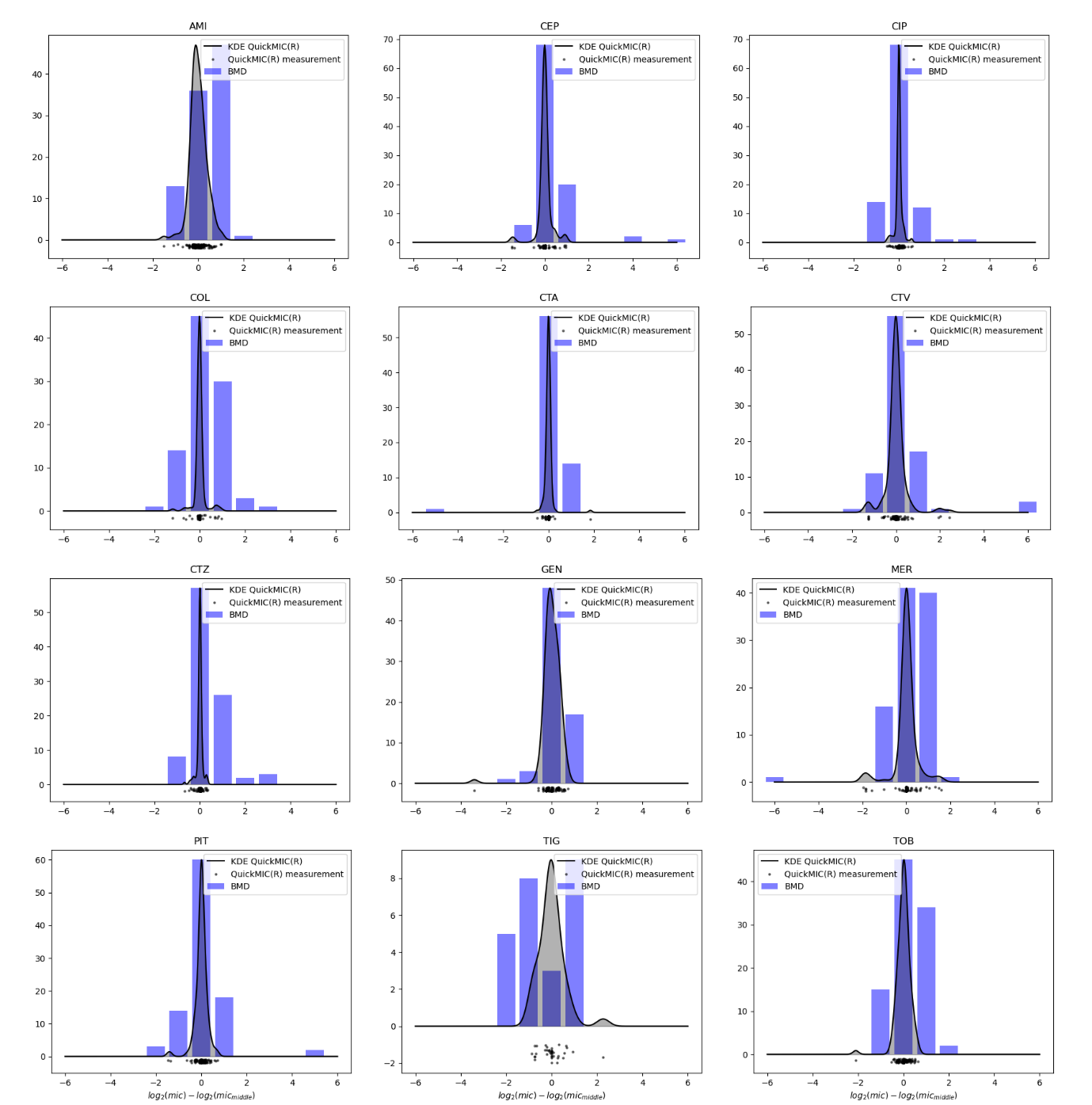


*Supplementary Figure 1. Comparison of measurement variability from BMD (blue bars) and QuickMIC linear MIC results ((black dots below the blue bars, and the grey shaded field = kernel density estimate (KDE) for visual comparison of spread)), measured in reproducibility isolates. . - The isolates were tested in triplicate and up to 14 times per antibiotic with the QuickMIC system at all four study locations. All MIC results are centred to the modal MIC for each drug/bug pair, meaning that each unit on the x-axis indicates one two-fold dilution result away from the modal MIC. Overall, QuickMIC results are less variable than BMD for all tested antibiotics.*


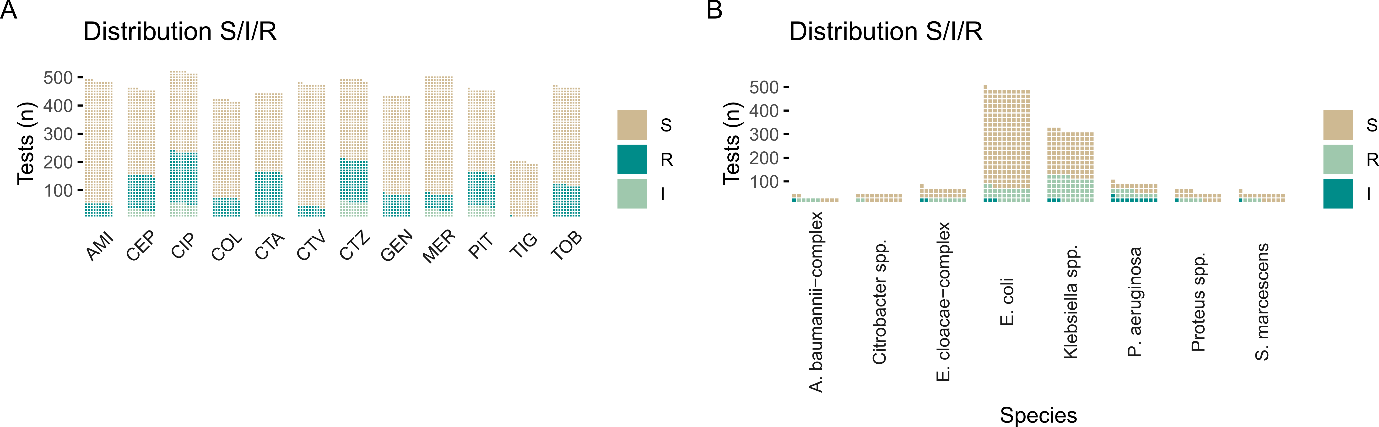


*Supplementary Figure 2. Distribution of susceptibility categories in the complete dataset, split over A) all included antibiotics and B) all included species.*

B

A


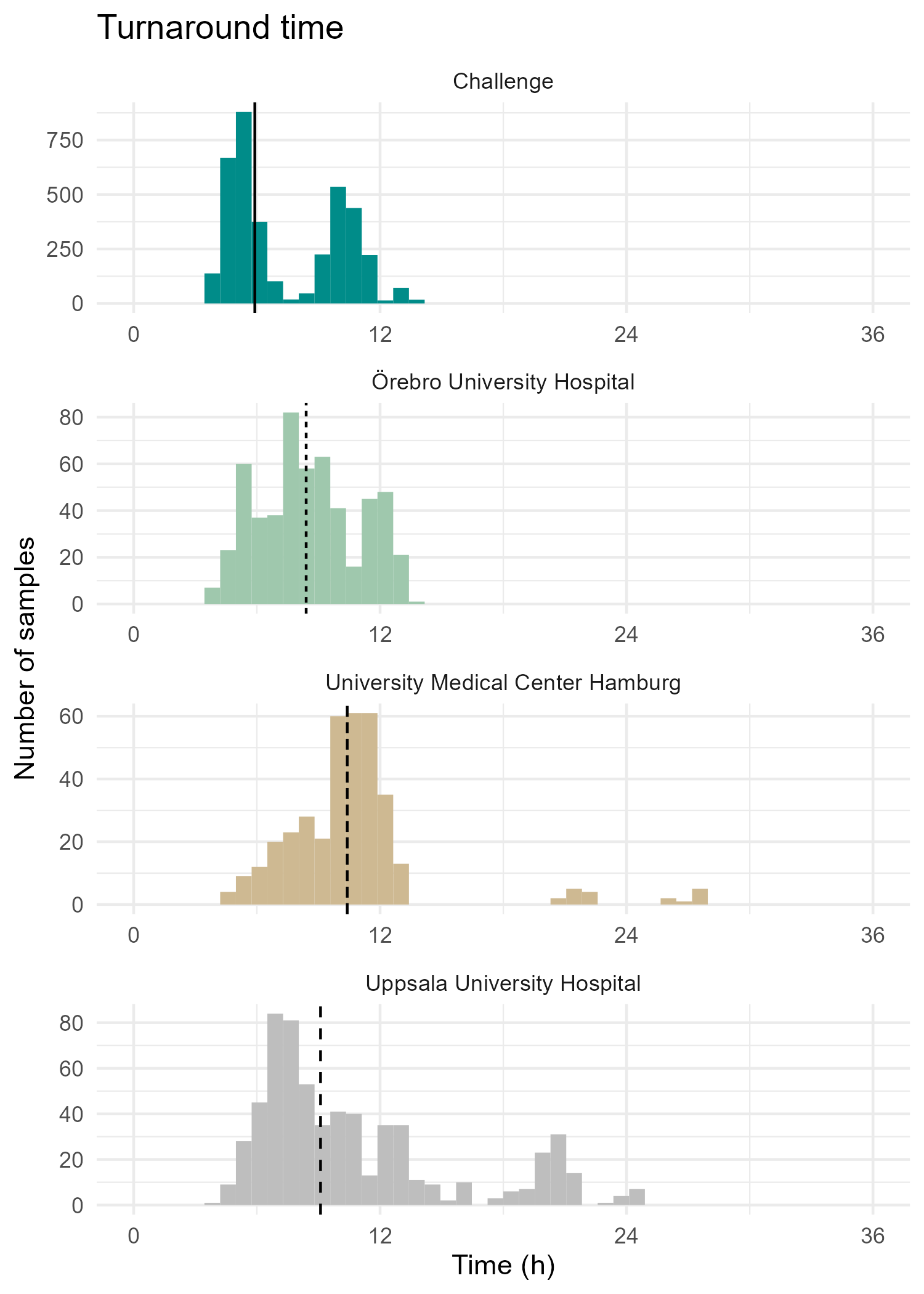

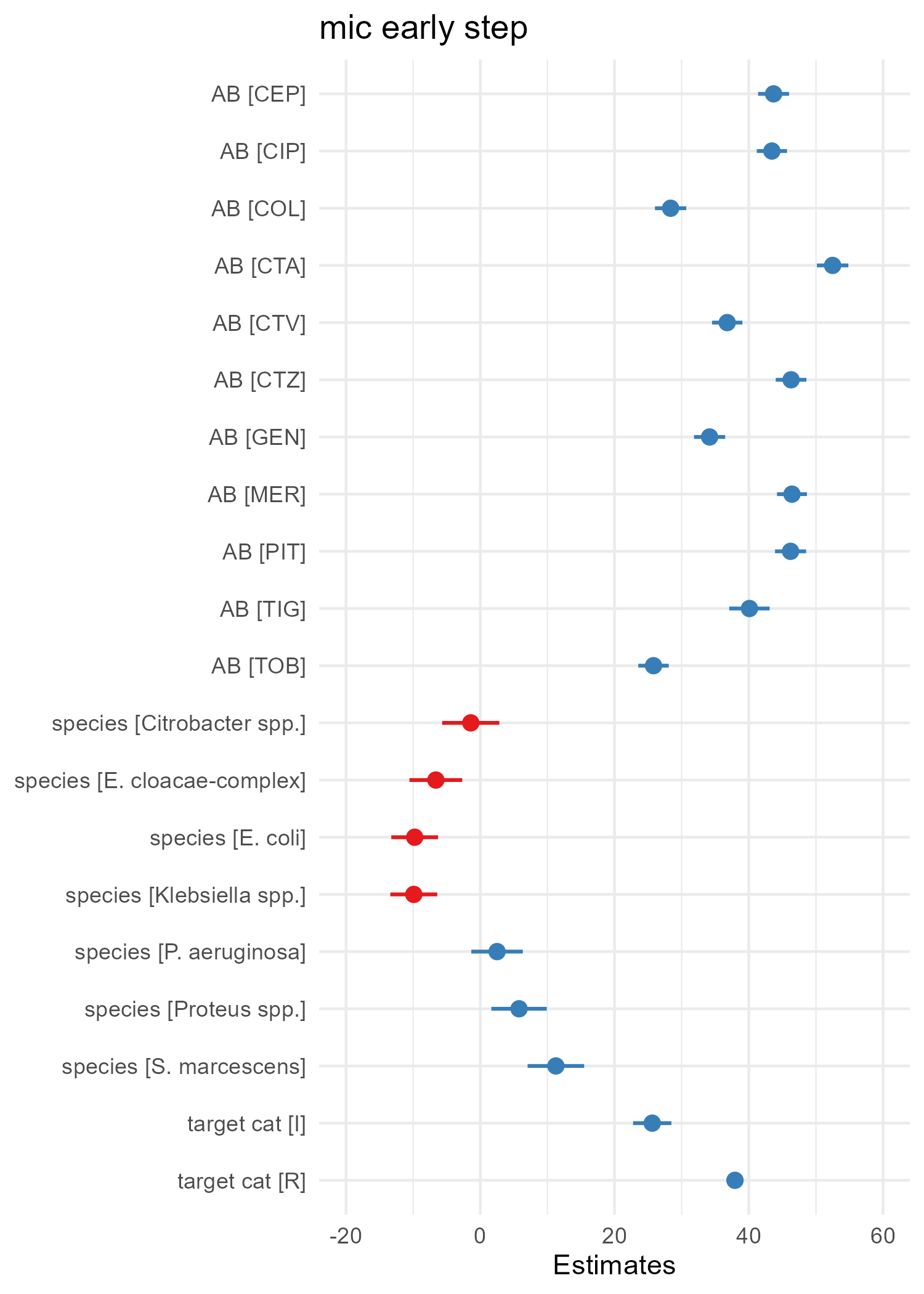


Supplementary Figure 3. A) Overview of turnaround time (TAT) from the different locations. Vertical black lines indicate mean values for each location. B) Estimated differences of QuickMIC analysis time from linear regression analysis, with contrasts set to Antibiotic (AB): AMI, species: Acinetobacter baumannii complex, Target category (cat): S. Red color indicate more rapid analysis time compared to the chosen contrast.
